# Corrigendum: RNAseq Profiling of Leukocyte Populations in Zebrafish Larvae Reveals a *cxcl11* Chemokine Gene as a Marker of Macrophage Polarization During Mycobacterial Infection

**DOI:** 10.3389/fimmu.2019.02720

**Published:** 2019-12-02

**Authors:** Julien Rougeot, Vincenzo Torraca, Ania Zakrzewska, Zakia Kanwal, Hans J. Jansen, Frida Sommer, Herman P. Spaink, Annemarie H. Meijer

**Affiliations:** ^1^Institute of Biology Leiden, Leiden University, Leiden, Netherlands; ^2^ZF-screens B. V., Leiden, Netherlands

**Keywords:** innate immunity, zebrafish, RNAseq, macrophage, mycobacteria, neutrophil, lymphoid progenitor cells

In the original article, there was an omission. The “**Data Availability Statement**” section was absent in the edited manuscript.

The **Data Availability Statement** has now been added to the published article:

“The sequencing data for infected samples have been submitted to the NCBI Gene Expression Omnibus (GEO; http://www.ncbi.nlm.nih.gov/geo/) under accession number GSE68920. The sequencing data for uninfected samples were made previously available under accession number GSE78954. The sequencing data for human macrophages are available under the accession number GSE36952.”

In the “Materials and Methods” section, “RNA Isolation, Illumina Sequencing, and Real time PCRs” subsection, the sequences of the primers ccr5(ccr12b.2)Fw and ccr5Rv were erroneous. The correct sequences are *ccr5*(*ccr12b.2*)*Fw*: GGCTTCCAACATCATCTTCACCCTCAC; *ccr5Rv*: CTATCATCCGAGTGCGCATGATGG.

The authors apologize for this error and state that this does not change the scientific conclusions of the article in any way. The original article has been updated.

