# Supplementary figures and images for: RNAseq Profiling of Leukocyte Populations in Zebrafish Larvae Reveals a cxcl11 Chemokine Gene as a Marker of Macrophage Polarization During Mycobacterial Infection
Source: Front Immunol. 2019 Apr 17;10:832. doi: 10.3389/fimmu.2019.00832 (PMC6499218; doi:10.3389/fimmu.2019.00832)

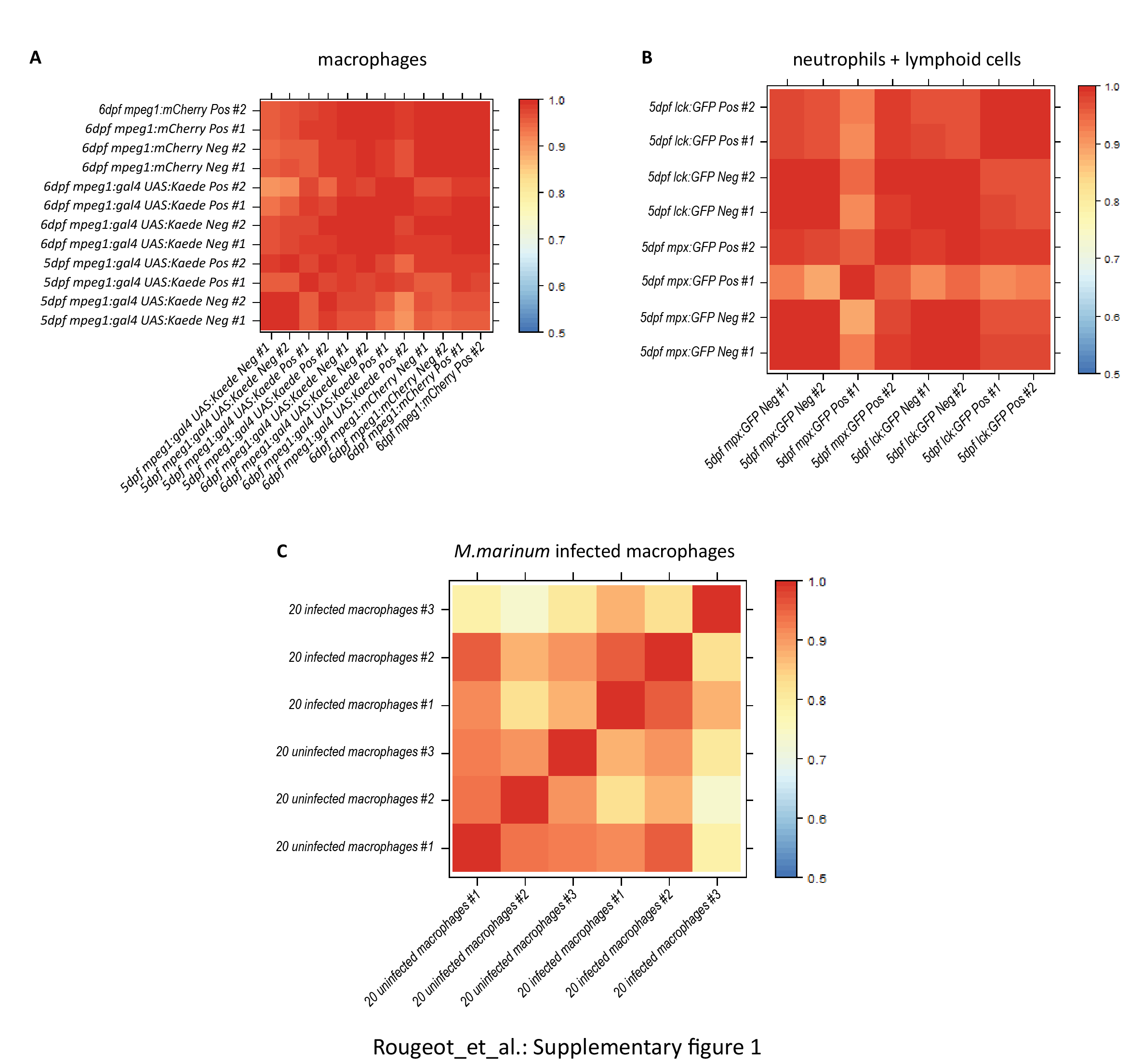

Supplement: Figure S1 — Correlation between RNA-sequencing samples. (A–C) HeatMap of Pearson correlation coefficient of 5 and 6 dpf mpeg1:gal4; UAS-Kaede, and mpeg1:mCherry positive and negative samples (A), 5dpf mpx:gfp and lck:gfp positive and negative samples (B) and 20 cell samples of mpeg1:gfp positive cells infected or not with M. marinum GFP (C). [file Image_1.tif]

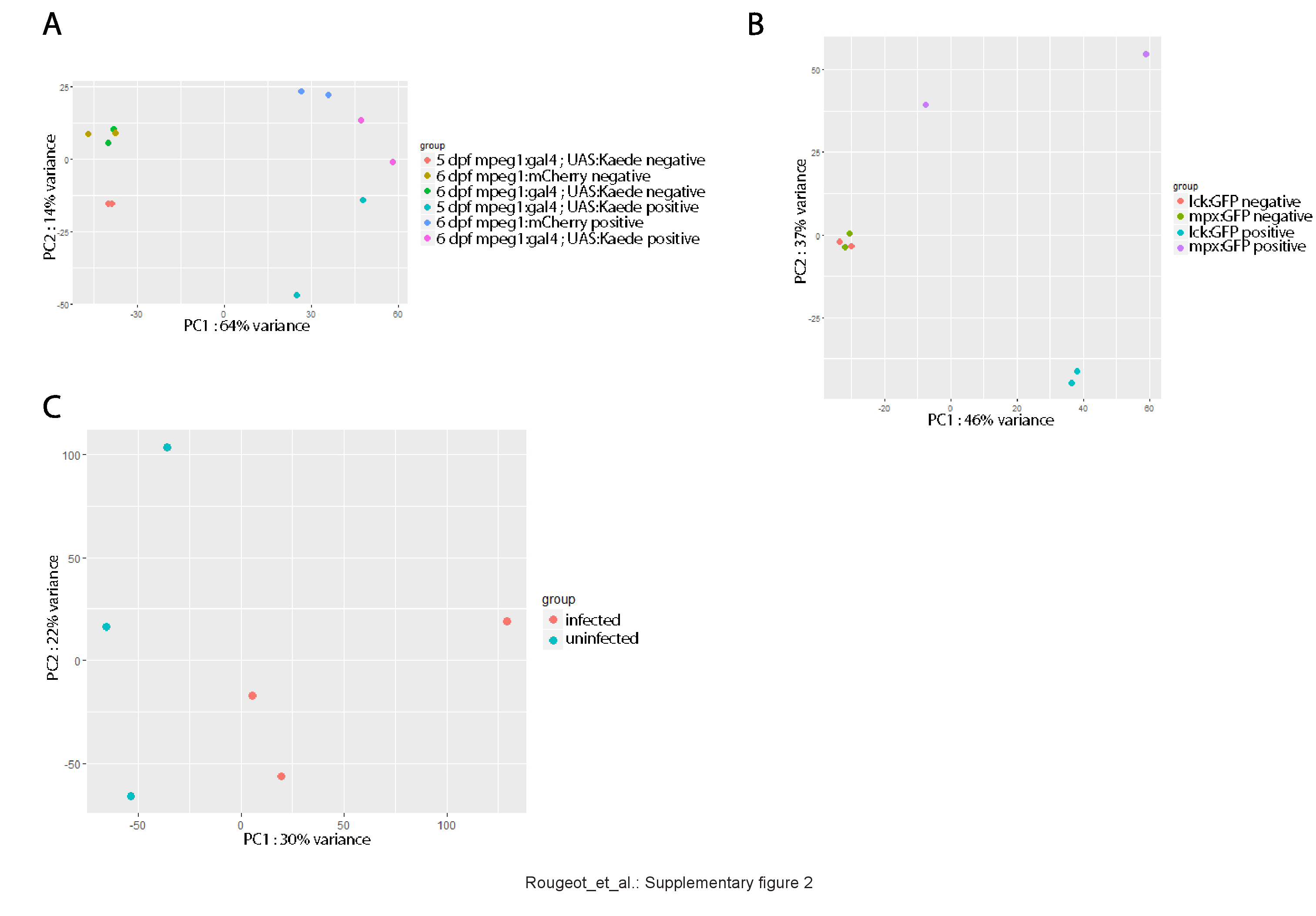

Supplement: Figure S2 — Principal Component Analysis of RNA-sequencing samples. (A–C) Principal Component Analysis of 5 and 6 dpf mpeg1:gal4; UAS-Kaede, and mpeg1:mCherry positive and negative samples (A), 5dpf mpx:gfp and lck:gfp positive and negative samples (B), and 20 cell samples of mpeg1:gfp positive cells infected or not with M. marinum GFP (C). [file Image_2.TIFF]

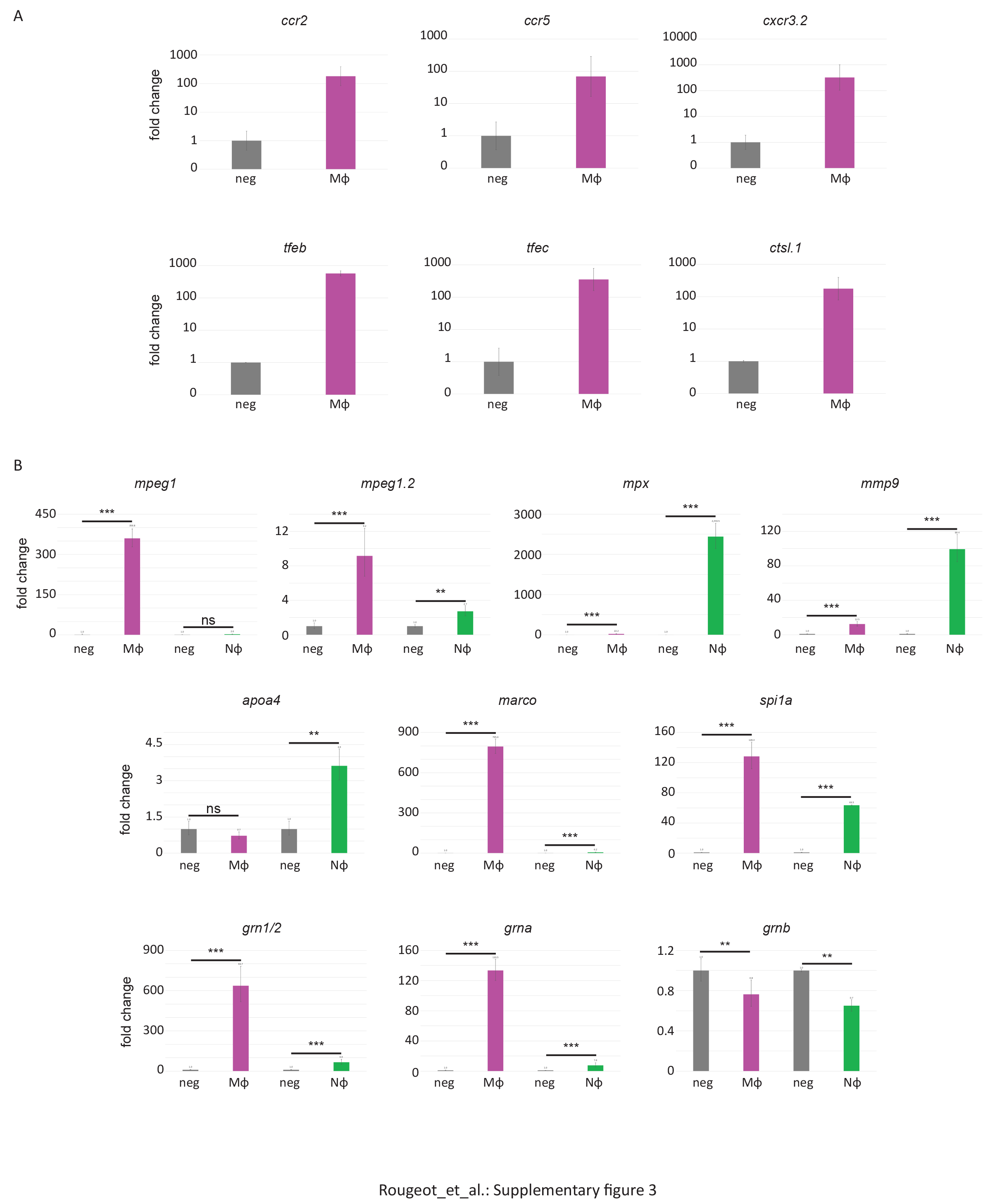

Supplement: Figure S3 — RT-qPCR validation of macrophage and neutrophils markers by RT-qPCR. (A) Expression of chemokine receptors and lysosomal genes in FACS-sorted macrophages (MΦ, mpeg1:mCherry positive) and compared with non-fluorescent cells (neg). All genes tested are present in the zf core dataset. RT-qPCR were performed in two independent biological replicates. Y-axis is log10-transformed for ease of visualization. (B) Expression of macrophage and neutrophil marker genes in FACS-sorted macrophages (MΦ, mpeg1:mCherry positive) and neutrophils (NΦ, mpx:GFP positive), and compared with their respective non-fluorescent cells (neg). The genes mpeg1, mpeg1.2, marco, spi1a, grn1/2, and grna were found enriched in the macrophage transcriptome dataset, mpx and mmp9 were found in both macrophage and neutrophil datasets, whereas apoa4 and grnb were found in none of them. RT-qPCR results are similar to RNA-seq results, however neutrophils qPCR results provide more significantly enriched genes. RT-qPCR were performed in three independent biological replicates. Statistical significance was analyzed by one-way ANOVA with Bonferroni post-hoc correction on ΔCt before transformation. Significance (P-value) is indicated with: ns, non-significant; *P < 0.05; **P < 0.01; ***P < 0.001. Error bars: mean ± s.e.m. [file Image_3.TIF]

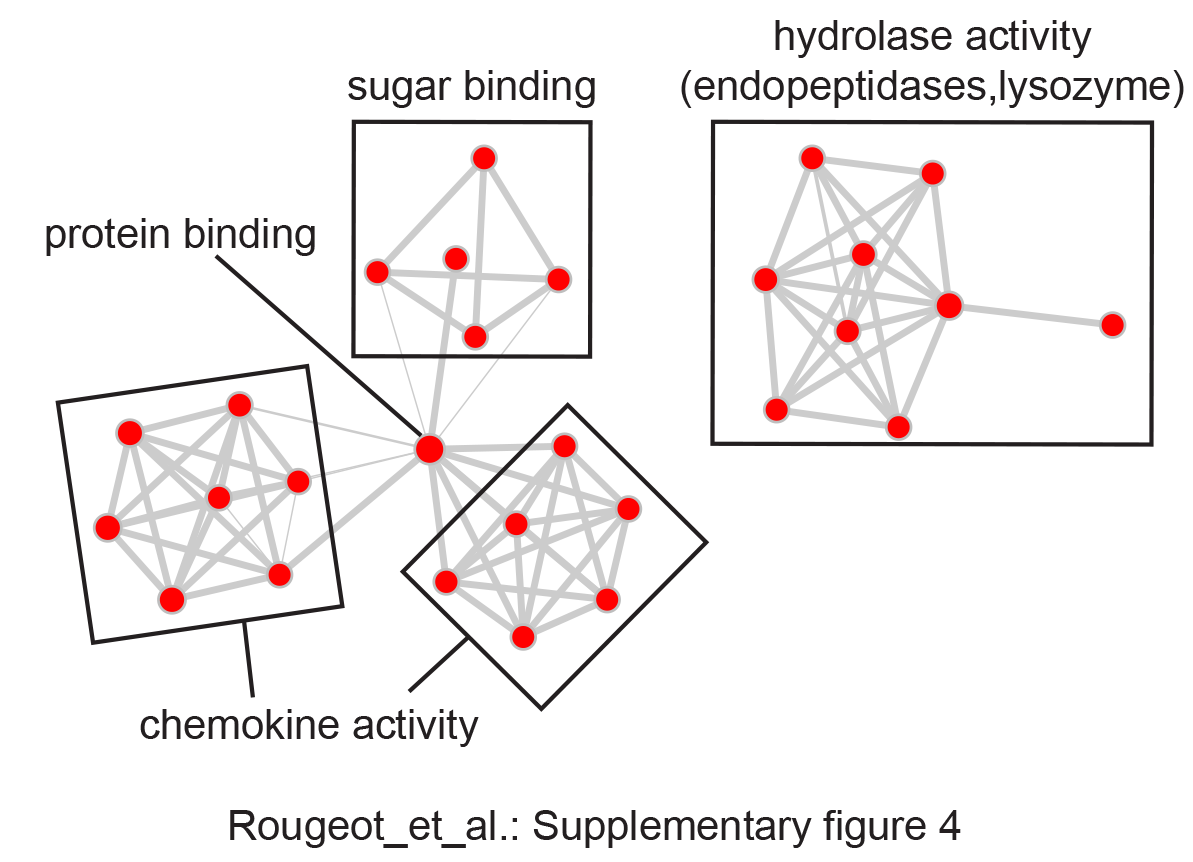

Supplement: Figure S4 — Molecular function associated to the zebrafish core macrophage expression dataset. Network visualization of GO analysis enrichment (molecular function category) of genes from the core macrophage expression data set using BiNGO and EnrichmentMap. Node size corresponds to the number of genes associated to the enriched GO term and edge size to the similarity coefficient between two nodes. [file Image_4.TIF]
